# Fibroblast Growth Factor 22 Inhibits ER Stress-Induced Apoptosis and Improves Recovery of Spinal Cord Injury

**DOI:** 10.3389/fphar.2020.00018

**Published:** 2020-02-11

**Authors:** Sipin Zhu, Mengji Chen, Min Chen, Jiahui Ye, Yibo Ying, Qiuji Wu, Haicheng Dou, Liyunian Bai, Fangmin Mao, Wenfei Ni, Kehe Yu

**Affiliations:** ^1^ Department of Orthopaedics, The Second Affiliated Hospital and Yuying Children’s Hospital of Wenzhou Medical University, Wenzhou, China; ^2^ Second Medical College of Wenzhou Medical University, Wenzhou, China

**Keywords:** spinal cord injury, fibroblast growth factor 22, ER stress, apoptosis, nerve regeneration

## Abstract

Currently, inhibiting or reducing neuronal cell death is the main strategy to improve recovery of spinal cord injury (SCI). Therapies using nerve growth factors to treat SCI mainly focused on reducing the area damaged by postinjury degeneration to promote functional recovery. In this report, we investigated the mechanism of ER (endoplasmic reticulum) stress-induced apoptosis and the protective action of fibroblast growth factor 22 (FGF22) *in vivo*. Our results demonstrated that ER stress-induced apoptosis plays a significant role in injury of SCI model rats. FGF22 administration promoted recovery and increased neuron survival in the spinal cord lesions of model mice. The protective effect of FGF22 is related to decreased expression of CHOP (C/EBP-homologous protein), GRP78 (glucose-regulated protein 78), caspase-12, X-box binding protein 1 (XBP1), eukaryotic initiation factor 2α (Eif-2α) and Bad which are ER stress-induced apoptosis response proteins. Moreover, FGF22 administration also increased the number of neurons and the expression of growth-associated protein 43 (GAP43) which was related to axon regeneration. We also demonstrated that the protective effect of FGF22 effectively reduces neuronal apoptosis and promotes axonal regeneration. Our study first illustrated that the function of FGF22 is related to the inhibition of ER stress-induced cell death in SCI recovery *via* activation of downstream signals. This study also suggested a new tendency of FGF22 therapy development in central neural system injuries, which involved chronic ER stress-induced apoptosis.

## Introduction

Spinal cord injury (SCI) is a destructive event that usually leads to significant functional impairment for the patient (Angeli et al., 2018). It triggers very limited regeneration in humans, leading to irreversible damage that can result in permanent motor dysfunction (Kumamaru et al., 2018; Sofroniew, 2018). SCI pathology can be divided into two stages: primary injury caused by direct disruption of the spinal cord, and secondary injury including neuronal apoptosis, autophagy, vascular dysfunction, oxidative stress and inflammation, which lead to sustained and extensive tissue damage (Tohda and Kuboyama, 2011; Zhu et al., 2016; He et al., 2017a). SCI causes the injury loci to form a hypoxic microenvironment, which in turn causes neuronal death and dysfunction, ultimately limiting function recovery after SCI (Sabelstrom et al., 2013). Therefore, SCI treatment focuses on reducing neuronal death by inhibiting neuronal apoptosis.

The endoplasmic reticulum (ER) was initially identified as an intracellular organelle responsible for maintaining cellular homeostasis and resisting potential injury caused by misfolded proteins on the ER (Zhang et al., 2015; He et al., 2017b). During SCI, a large amount of protein misfolding caused by alteration of the microenvironment in the injured area leads to the unfolded protein response (UPR) (Hetz, 2012). The UPR activates three signal pathways that are regulated by activating transcription factor 6 (ATF6) and protein kinase RNA-like endoplasmic reticulum kinase (PERK), which increases the expression of apoptotic proteins and leads to neuronal death (Lee et al., 2014; Dou et al., 2018). Extensive neuronal death induced by apoptosis is the largest obstacle in recovery of spinal cord injury (Crowe et al., 1997).

The family of fibroblast growth factors and their receptors have been important regulators of presynaptic differentiation. FGF22 is a member of the FGF7 subfamily. It mainly activates FGF receptors FGFR1b and FGFR2b (Umemori et al., 2004). Studies have shown that FGF22 plays a significant part in SCI recovery as an endogenous regulator of synaptic plasticity and circuit remodeling in the adult nervous system (Jacobi et al., 2015). In this study, we injected different concentrations of FGF22 into the site of SCI in rats to observe the recovery of injured spinal cord. Here, we show that FGF22 can inhibit neuronal apoptosis induced by ER stress and promote SCI recovery.

## Materials and Methods

### Cell Culture and Treatment

PC-12 cell lines obtained from the American Type Culture Collection (ATCC) were cultured in RPMI 1640 medium, which consisted of 10% fetal bovine serum (FBS), RPMI 1640 and 1% antibiotics. Cell incubation was performed in a humidified incubator at 37°C and 5% CO_2_. All cells were randomly divided into four groups which included the 4-PBA (4-phenylbutyric acid) group, TG (thapsigargin) group, control group, treatment group with 5 μg/ml FGF22, treatment group with 10 μg/ml FGF22 and treatment group with 15 μg/ml FGF22.

### *In Vitro* Scratch Motility Assay

A linear scratch was applied to cell monolayers with the help of a 200 µl pipette tip to create a cell-free area; then, cells were washed twice with PBS. Wound analysis and photography were performed by an inverted phase-contrast microscope and the wound area was calculated by ImageJ software.

### Apoptosis Assay

The apoptotic rates of the PC-12 cells treated with TG, TG + 4-PBA, TG+5 μg/ml FGF22, TG + 10 μg/ml FGF22 and TG + 15 μg/ml FGF22 were measured by the PI/Annexin V-FITC kit (Invitrogen, Carlsbad, CA, USA) and then analyzed by FACS can flow cytometer (Becton Dickinson, Franklin Lakes, NJ, USA) as the manual description.

### Western Blot Analysis

For protein analysis, PC12 cells were in RIPA buffer (25 mM Tris-HCl, 150 mM NaCl, 1% Nonidet P-40, 1% sodium deoxycholate, and 0.1% SDS) containing the protease and phosphatase inhibitor. Cracked. The above extracts were quantified using bicinchoninic acid (BCA) reagent (Thermo, Rockford, IL, USA). 50 μg of protein was placed on the 11.5% gel and then transferred to a PVDF membrane (Bio-Rad, Hercules, CA, USA). The membrane was blocked with 5% milk (Bio-Rad) in TBS containing 0.05% Tween 20 for 1 h and incubated with the following antibodies: CHOP (1: 300), GRP78 (1:300), caspase-12 (1:1000), and GAPDH (1:1000). The membrane was washed 3 times with TBS and treated with a horseradish peroxidase-conjugated secondary antibody for 1 hour at room temperature. The signals were visualized by the ChemiDoc ™ XRS+ imaging system (Bio-Rad), and the band density was quantified using the Multigauge Software (FUJIFILM Corporation, Tokyo, Japan) of the 2006 Scientific Laboratory. We analyzed the relative density of the bands using quantity one (version 4.5.2; Bio-Rad).

### Animal Model of Spinal Cord Injury

Eighty adult female SD rats (weighing 220–250 g at the start of the experiment) were purchased from the Animal Center of Chinese Academy of Sciences, Shanghai, China. The Animal Care and Use Committee of Wenzhou Medical College ratified the experiments, and the experiments were carried out in accordance with the guidelines of the Care and Use of Laboratory Animals from the National Institutes of Health. Animals were randomly divided into four groups, which included the SCI group, sham group, treatment group with 5 μg/ml FGF22 and treatment group with 10 μg/ml FGF22. The animals were fixed with a skin incision along the midline of the back. The 8^th^-to-10^th^ thoracic spinal vertebrae were exposed. The model of acute spinal cord injury was established by striking T9 segments of the spinal cord with a 10-g hammer and a 25-mm-height free fall. The sham group rats underwent the operation similarly without injury by collision. Animal care and handling involved bladder massage to induce urination twice daily, once in the morning and again in the evening, until cefazolin sodium reconstructed reflex bladder function (50 mg/kg, i.p.).

### FGF22 Transplantation to Treat SCI

After building the SCI model, surgery was carried out immediately to microinject 5 μg/ml and 10 μg/ml of FGF22 into the rats of the two FGF22 treated groups. Through stereoscopic positioning instruments and microsyringes, FGF22 orthotopically arrived at the injured lesion. The sham group was perfused with saline at the same site. All animals were returned to cages for recovery. We provided each group with the same moderate diet at fixed times.

### Locomotion Recovery Assessment

To examine the locomotor function of rats after SCI, behavioral analyses were conducted by two well-trained investigators familiar with the score criteria but blinded to the experimental conditions.

Basso-Beattie-Bresnahan (BBB) is a scale with a total score of 22 points (scores 0–21) that logically and systematically follows functional recovery of hindlimbs from 0 points, reflecting complete paralysis of the lower limbs, to 21 points, reflecting normal locomotor function (Basso et al., 1995). The scale was formulated based on the natural progression of motor recovery in rats with thoracic spinal cord injury.

The inclined plane test was carried out by means of a testing apparatus (Rivlin and Tator, 1977). The maximum angle in which a rat’s position could be maintained for 5 s without falling was recorded for each position, and then, the averages were taken to determine each rat’s scores. Footprint analysis was performed by dipping the animal’s hind paws in red dye and allowing them to crawl past a box of suitable size (1 m long and 7 cm wide) (de Medinaceli et al., 1982). Footprint scanning and digital image analysis were performed.

### H&E Staining and Nissl Staining

The rats were re-anesthetized with 1% pentobarbital (40–50 mg/kg, i.p.). Thoracotomy was performed on the 60th day after injection. Rats were perfused with 0.9% NaCl, followed by 500 ml paraformaldehyde phosphate buffer solution injected into the heart to harden the tissue for complete removal. Spinal cord excision at the 8^th^-to-10^th^ thoracic spinal vertebral level around the injury was performed. Spinal cords were fixed overnight in cold 4% paraformaldehyde and embedded with paraffin. For histopathological examination, transverse paraffin sections (10 μm thick) were subjected to hematoxylin and eosin (H&E) staining. For Nissl staining, sections were incubated in 1% cresyl violet. Two stained sections were examined and scanned under a light microscope.

### Immunohistochemistry

Transverse paraffin sections were incubated in 80% carbinol and 3% H_2_O_2_ for 30 min, and then, the sections were transferred to blocking solution at room temperature for 1 h. Subsequently, the sections were incubated at 4°C overnight with the following primary antibodies: GRP78 (1:200), CHOP (1:200), caspase-12 (1:4000), XBP1 (1:400), Eif-2α (1:100) and Bad (1:200). After washing with PBS three times, sections were incubated with horseradish peroxidase-conjugated secondary antibodies at 37°C for 2h. We used 3,3-diaminobenzidine (DAB) to stop the reaction. Positive neuron numbers and optical densities of CHOP, GRP78, XBP1, Eif-2α, Bad and caspase-12 were counted in six randomly selected fields per sample. The results were imprinted using Nikon ECLPSE 80i (Nikon, Tokyo, Japan).

### Video Images Locomotor Function

Forty-two adult female SD rats (weighting 220–250 g) are divided into six groups, sham group, SCI group, TG group, 4-PBA group, 5 μg/ml FGF22 group and 10 μg/ml FGF22 group. Using a camera (Leica), each group rats were photographed while walking through a 1-m-long glass runway with markers on the hindlimbs to estimate hip, knee, ankle, and foot position. The following parameters were used to evaluate locomotion: 1) weight support (height; hip height minus trunk width, equal to the torso gap on the ground), 2) leg extensor spasms (quantified as the time the foot is overstretched and dragged, relative to the foot cycle The duration is on the back surface), 3) the number of footsteps (the number of footsteps calculated relative to the number of forefoot steps) and 4) the posture of the foot (measurement of the foot offset behind the hip at the beginning of the ankle). The walking step rhythm is defined by the front leg (front leg steps/sec).

### Immunofluorescence Staining

Sections were incubated with 10% normal bovine serum at 37°C for 1 h in PBS containing 0.1% Triton X-100 and then incubated with suitable primary antibodies overnight in the same buffer solution at 4°C. Nuclei were stained with DAPI (0.25 μg/ml) dye. To detect neurons and GAP43, we used anti-NeuN (1:500, Millipore) and anti-GAP43 (1:50) primary antibodies, respectively. After incubation with primary antibody, sections were triple washed with PBS at room temperature, followed by incubation with secondary antibody (1:500) at 37°C for 1 h. Then, sections were washed with PBS containing 0.1% Triton X-100 4×10 min and then washed with PBS 3×5 min. All images were captured under a Nikon ECLIPSE Ti microscope (Nikon, Tokyo, Japan).

### Statistical Analysis

Statistically, data were expressed as the mean ± SEM. For two experimental groups, the Student’s *t* test was used to determine statistical significance. Values of *P* ≤ 0.05 were deemed significant. When there were more than two experimental groups, one-way analysis of variance (ANOVA) and Dunnett’s *post hoc* test were used to statistically evaluate data, and *P* ≤ 0.05 was considered statistically significant.

## Results

### FGF22 by Inhibiting the Endoplasmic Reticulum Stress Increases the Migration and Repair Capability of PC-12 Cells

**Figure 1:**
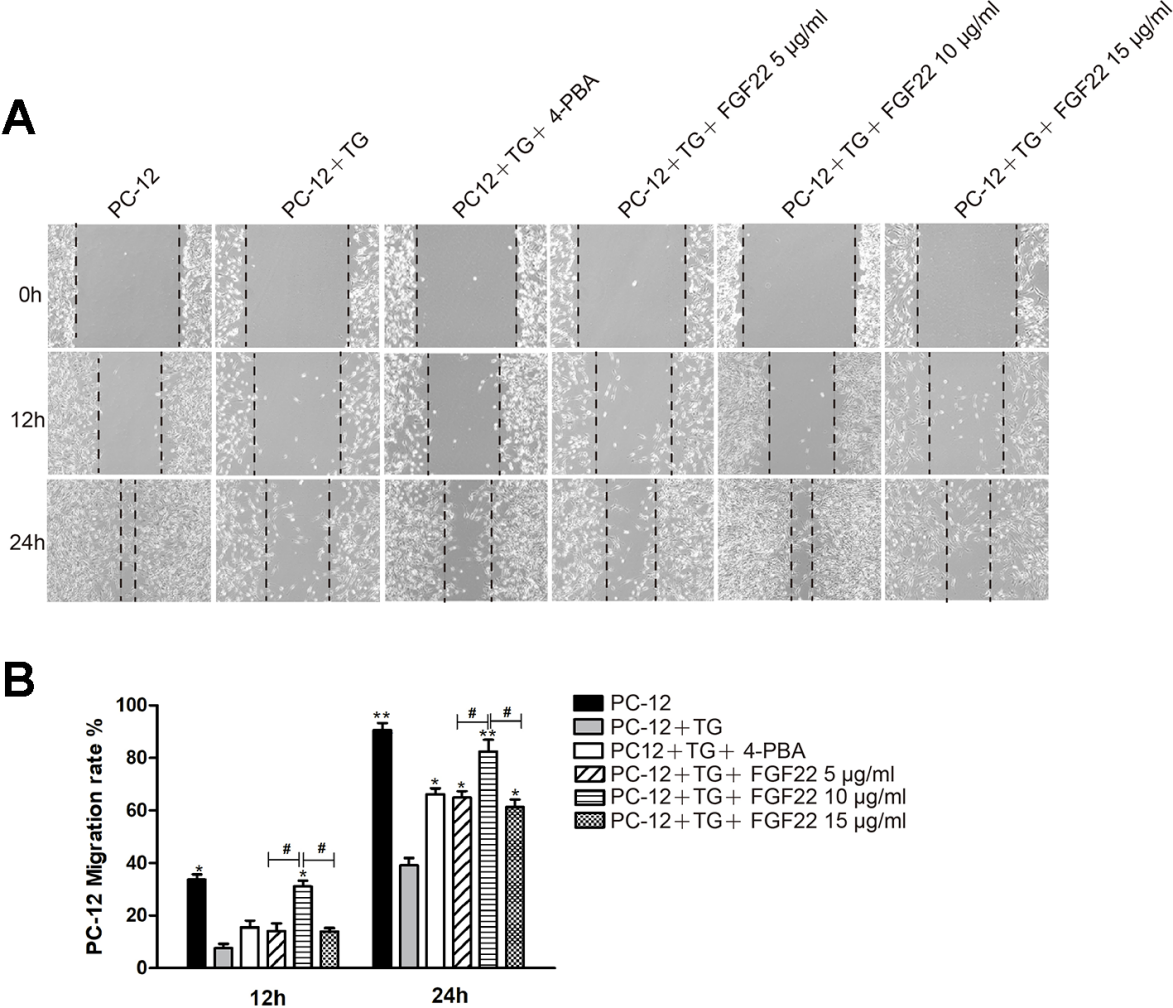
FGF22 by inhibiting the endoplasmic reticulum stress promoted PC-12 cell migrate. **(A)** Images of wound healing in the control group, TG group, TG + 4-PBA group, TG + 5 μg/ml FGF22 group, TG + 10 μg/ml FGF22 group and TG + 15 μg/ml FGF22 group. These images were captured by an inverted phase-contrast microscope at 0, 12, and 24 h after the scratch. **(B)** The PC-12 migration rate that affected by the above factors for 12 h and 24 h. The data was acquired by using Image J 1.38 and were expressed as the mean values ± SEM, *n* = 3. “*”*p* < 0.05, “**” *p* < 0.01 vs PC-12+TG group and “^#^” represent *P* < 0.05 comparing the 5 μg/ml FGF22 group and the 15 μg/ml FGF22 group with the 10 μg/ml FGF22 group separately indicating statistical significance.

PC12 cells were derived from murine adrenal medullary pheochromocytoma, and were similar to normal nerve cells in terms of cell morphology, physiology, biochemistry and other functions (Shafer and Atchison, 1991). Wound healing assay results were shown for the control group, TG group, 4-PBA group, 5 μg/ml FGF22 group, 10 μg/ml FGF22 group and 15 μg/ml FGF22 group 12 h and 24 h after scratching (**Figure 1A**). Compared to the control group, the TG group displayed a larger cell-free area, and compared with the TG group, the 10 μg/ml FGF22 treatment group displayed an obviously reductive wound area and faster migration. Although the 4-PBA group, the 5 μg/ml FGF22 group and the 15 μg/ml FGF22 group had improved cell migration, the 10 μg/ml FGF22 treatment group was even more improved (**Figure 1B**). The effect of the 15 μg/ml FGF22 group was not as good as that of the 10 μg/ml FGF22 group, which may be due to the fact that FGF22 was the biological macromolecule. If the concentration was too high, the cells will dehydrate and the cell migration capacity will be reduced (Yurinskaya et al., 2005).

### FGF22 Inhibit ER Stress-Induced Cell Apoptosis of PC-12 Cells

**Figure 2:**
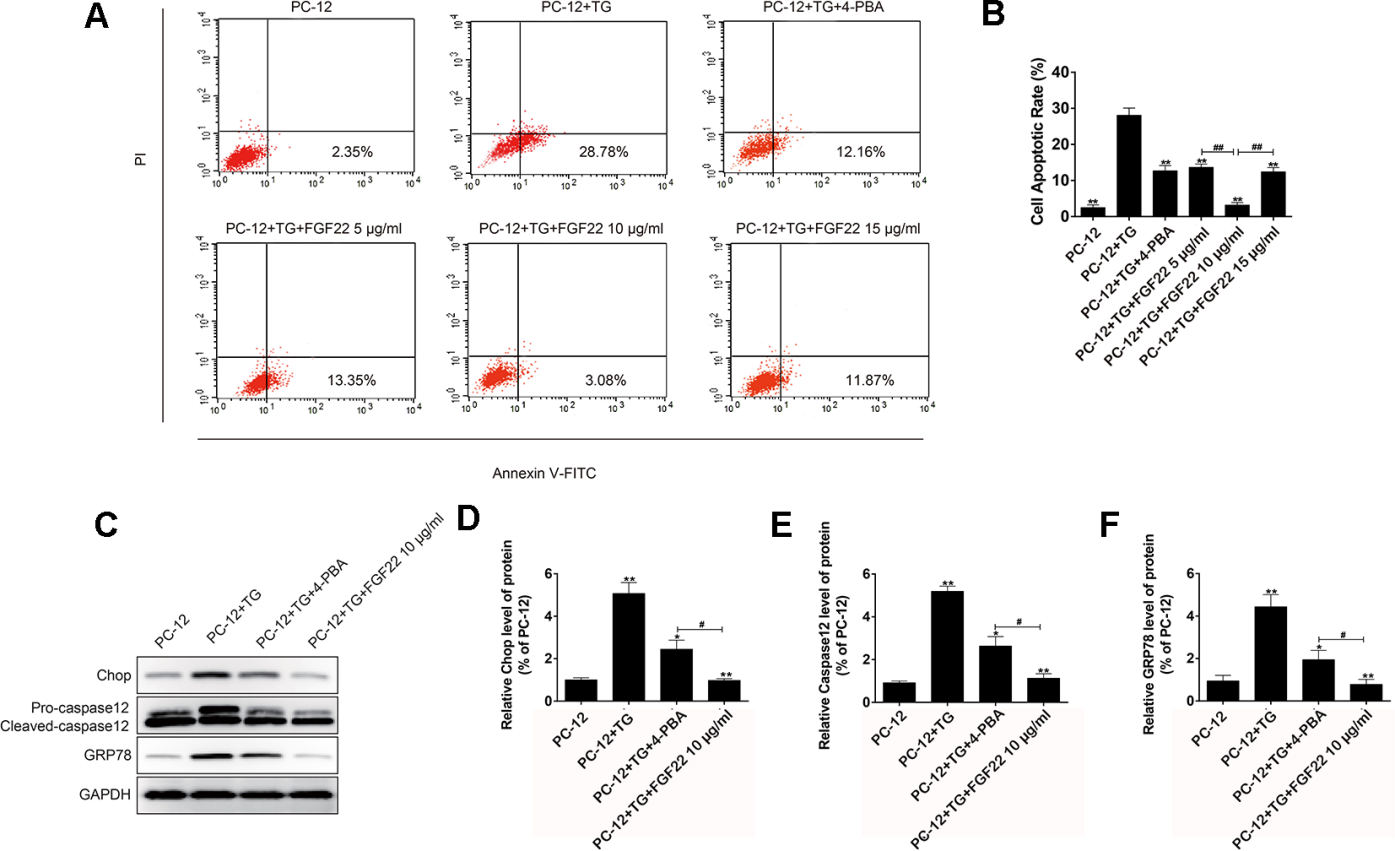
FGF22 inhibit ER stress-induced cell apoptosis. **(A)** FACS can result from PI/annexin V-FITC staining for cell apoptosis analysis. **(B)** Statistical result of apoptosis rate in PC12 cells treated with TG, TG + 4-PBA, TG+5 μg/ml FGF22, TG + 10 μg/ml FGF22 and TG + 15 μg/ml FGF22. “**”*p* < 0.01 versus the TG treatment group and “^##^” *P* < 0.01 indicating statistically significant. **(C)** The protein expressions of GRP78, CHOP, and caspase-12 in the control group, TG group, TG + 4-PBA group, TG + 10 μg/ml FGF22 group were tested with western blotting. GAPDH was used as the loading control and for band density normalization. **(D–F)** The optical density analysis of GRP78, CHOP, and caspase-12 protein. “*”*P* < 0.05 and “**”*P* < 0.01 versus the SCI group, “^#^” *P* < 0.05 indicating statistically significant.

To further confirm the role of FGF22 in the ER stress induced apoptosis *in vitro*. The apoptosis model was replicated by ER stress specific activator TG was using to treat PC12 cells. Cell apoptosis rates were analyzed, and we found that FGF22 10 ug/ml significantly reduced TG-induced apoptosis rate in PC12 cells, compared with the TG, TG + 4-PBA, FGF22 5 μg/ml and FGF22 15 μg/ml groups (**Figures 2A, B**). Furthermore, the ER stress-related protein levels were detected by western blot. We observed that the levels of CHOP, GRP78, and cleaved caspase-12 protein significantly decreased in FGF22 10 ug/ml group than other groups (*P* < 0.01) (**Figures 2C–F**).

### Maximizing Functional Recovery After SCI With a Higher Concentration of FGF22

**Figure 3:**
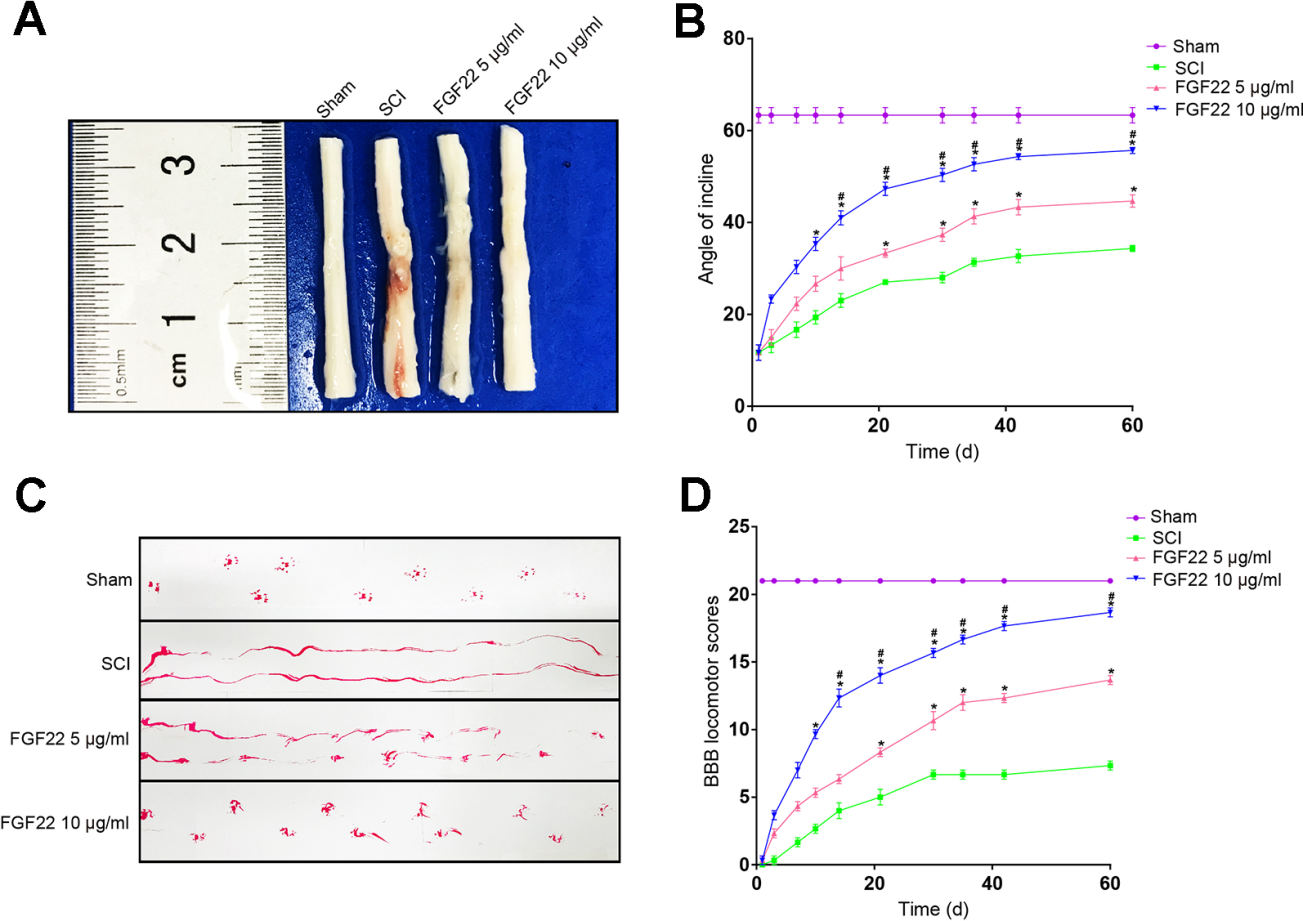
FGF22 improved the motor function of rats with SCI. **(A)** Anatomical features of the sham group, SCI group, 5 μg/ml FGF22 group and 10 μg/ml FGF22 group in spinal cord injury, 60 days after SCI. **(B)** The BBB scores of the sham group, SCI group, 5 μg/ml FGF22 group and 10 μg/ml FGF22 group. The score of the sham group was 21 points, which indicates normal locomotion. “*” represents *P* < 0.05 versus the SCI group, “^#^” represents *P* < 0.05 versus the 5 μg/ml FGF22 group. Data are the mean values ± SEM, *n* = 6. **(C)** The inclined plane test scores of the different groups. “*” represents *P* < 0.05 versus the SCI group, “^#^” represents *P* < 0.05 versus the 5 μg/ml FGF22 group. Data are the mean values ± SEM, *n* = 6. **(D)** Footprint analyses of the different groups at 60 days.

FGF22 treatment reduced spinal cord injury and neuron death and improved motor recovery after SCI. The FGF22 injection groups showed signiﬁcantly higher BBB scores than the SCI group after the operation, indicating that FGF22 promoted the recovery of nerve function. The results of the inclined plane test were similar. The 10 μg/ml FGF22 group showed the most complete anatomical appearance of SCI (**Figure 3A**). The mean BBB scores and angle of incline scores assessing locomotor skills over time were higher in the 10 μg/ml FGF22 group than in the other three groups (**Figures 3B, C**). Rats injected with 10 μg/ml FGF22 also showed a significant improvement in footprint analysis and at day 60 (**Figure 3D**).

### FGF22 Increases Neuron Survival and Improves Tissue Density in SCI

**Figure 4:**
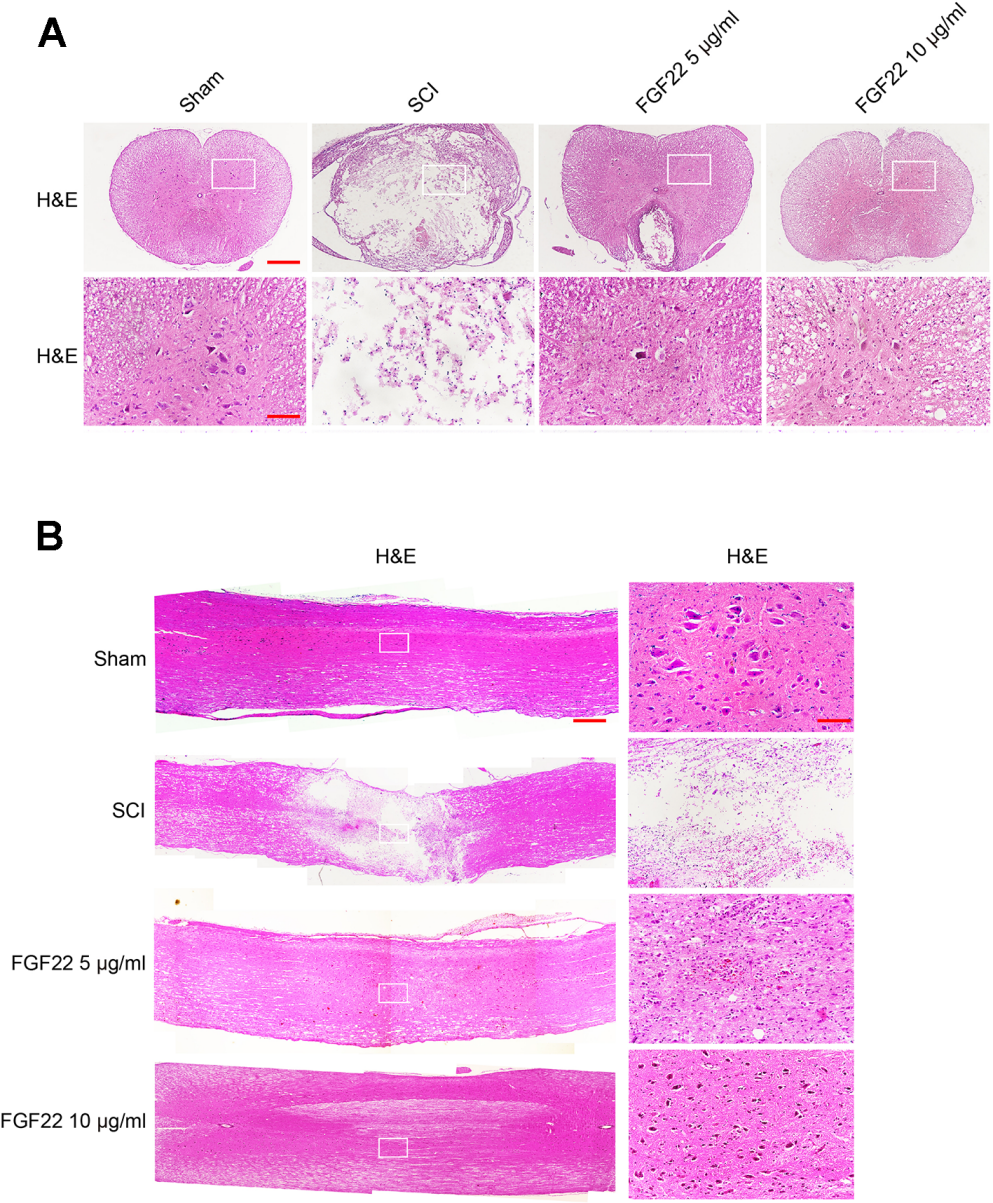
FGF22 improved the recovery of SCI. **(A)** H&E staining (cross-section) results for the sham, SCI group, 5 μg/ml FGF22 group and 10 μg/ml FGF22 group, scale bar = 500 μm. A boxed region illustrates a representative region with high power images, scale bar = 100 μm. **(B)** H&E staining (longitudinal section) results for the sham, SCI group, 5 μg/ml FGF22 group and 10 μg/ml FGF22 group, scale bar = 500 μm. A boxed region illustrates a representative region with high power images, scale bar = 100 μm.

Transverse and longitudinal H&E staining was performed on spinal cord samples in the sham group, SCI group, 5 μg/ml FGF22 group and 10 μg/ml FGF22 group after 60 days contusion (**Figures 4A, B**). Compared with the sham group, the SCI group showed progressive destruction of the dorsal white matter and central gray matter tissue. Compared with the SCI group, the 10 μg/ml FGF22 treatment group showed obvious protective impacts, such as less necrosis and karyopyknosis. Although the 5 μg/ml FGF22 group showed improvements in spinal cord sections, 10 μg/ml FGF22 treatment provided more amelioration (**Figures 4A, B**). These results further strengthen the neuroprotective efficacy of 10 μg/ml FGF22 for motor neurons in this SCI rat model.

### FGF22 Treatment Inhibits ER Stress-Induced Apoptosis and Improves Recovery of SCI

**Figure 5:**
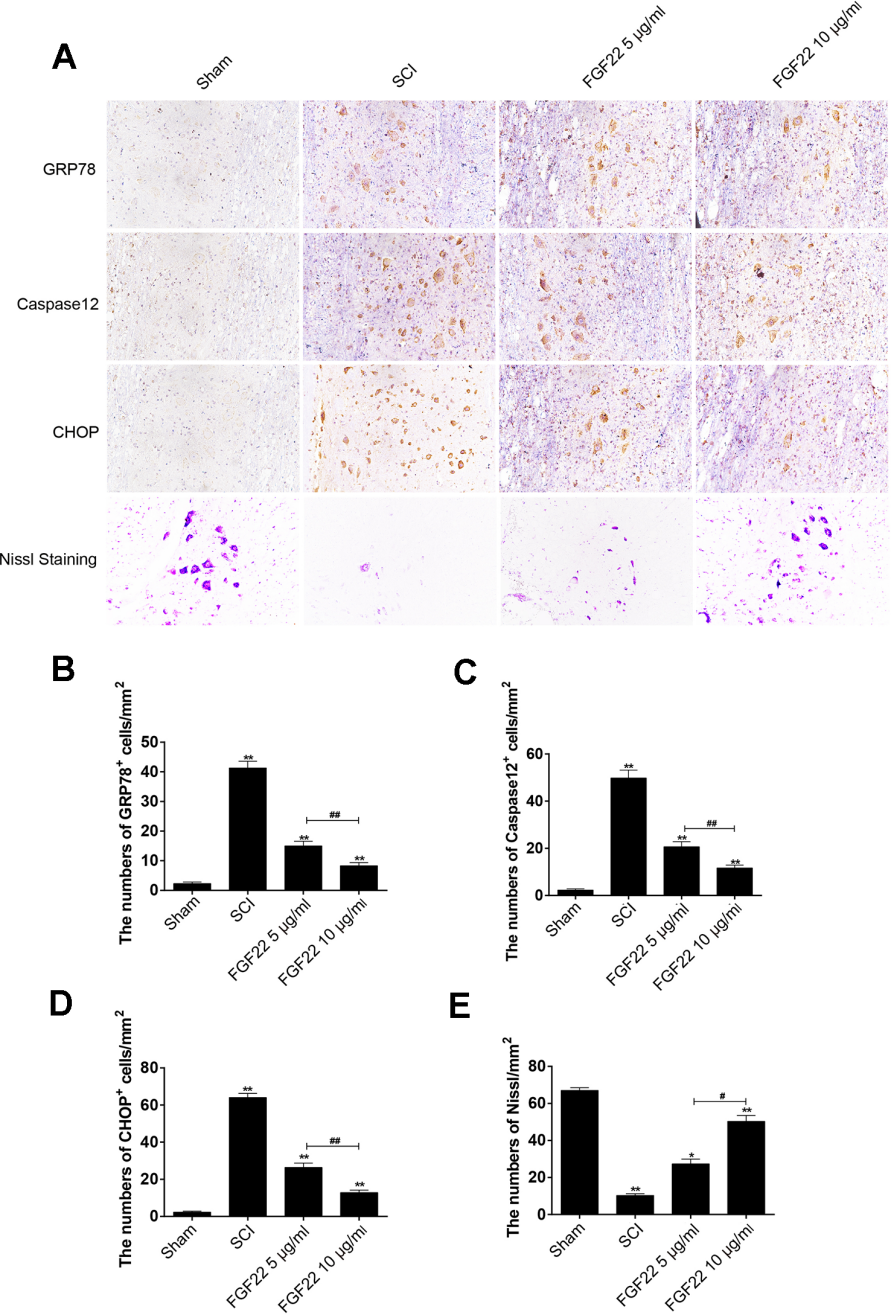
FGF22 inhibited ER stress-induced apoptosis and improves the recovery of SCI. **(A)** Immunohistochemistry for GRP78, CHOP, and caspase-12 in the sham, SCI, 5 μg/ml FGF22 and 10 μg/ml FGF22 groups. **(B)** Nissl staining of crosscutting for the sham, SCI, 5 μg/ml FGF22 and 10 μg/ml FGF22 groups. **(C**–**E)** Analysis of positive cells in immunohistochemistry and Nissl staining of crosscutting. “*” and “**” represent *P* < 0.05 or *P* < 0.01 versus the sham group or SCI group, “^#^” and “^##^” represent *P* < 0.05 or *P* < 0.01 comparing the 10 μg/ml FGF22 group to the 5 μg/ml FGF22 group, indicating statistical significance. Data are the mean values ± SEM, *n* = 6.

To determine whether FGF22 promoted SCI recovery *via* inhibiting ER stress-induced apoptosis, we measured the expression of GRP78, caspase-12 and CHOP, which are related to ER stress, by immunofluorescence staining. As shown in **Figure 5**, caspase-12, GRP78 and CHOP expression were increased in SCI and remarkably inhibited by FGF22. Moreover, the 10 μg/ml FGF22 group showed fewer positive points than the 5 μg/ml FGF22 group. This indicates that, within a certain range, higher concentrations of FGF22 can better inhibit apoptosis and promote recovery of SCI (**Figures 5A–D**).

After SCI, the lateral intumescent spinal cord anterior horn displayed a decreased number of large and medium-sized neurons compared with the sham group, the survival rate decreased, and the cell outline was not clear, as seen by fuzzy Nissl staining. Nissl staining of crosscutting showed that the 10 μg/ml FGF22 group recovered well and the spinal cord anterior horn neurons of Nissl increased compared to the SCI group, providing further evidence that spinal cord function restoration and the treatment effect were more pronounced in the 10 μg/ml FGF22 group than in the 5 μg/ml FGF22 group (**Figures 5A, E**).

### Regulation of Upstream and Downstream Signals Is Important for the Protection of FGF22

**Figure 6:**
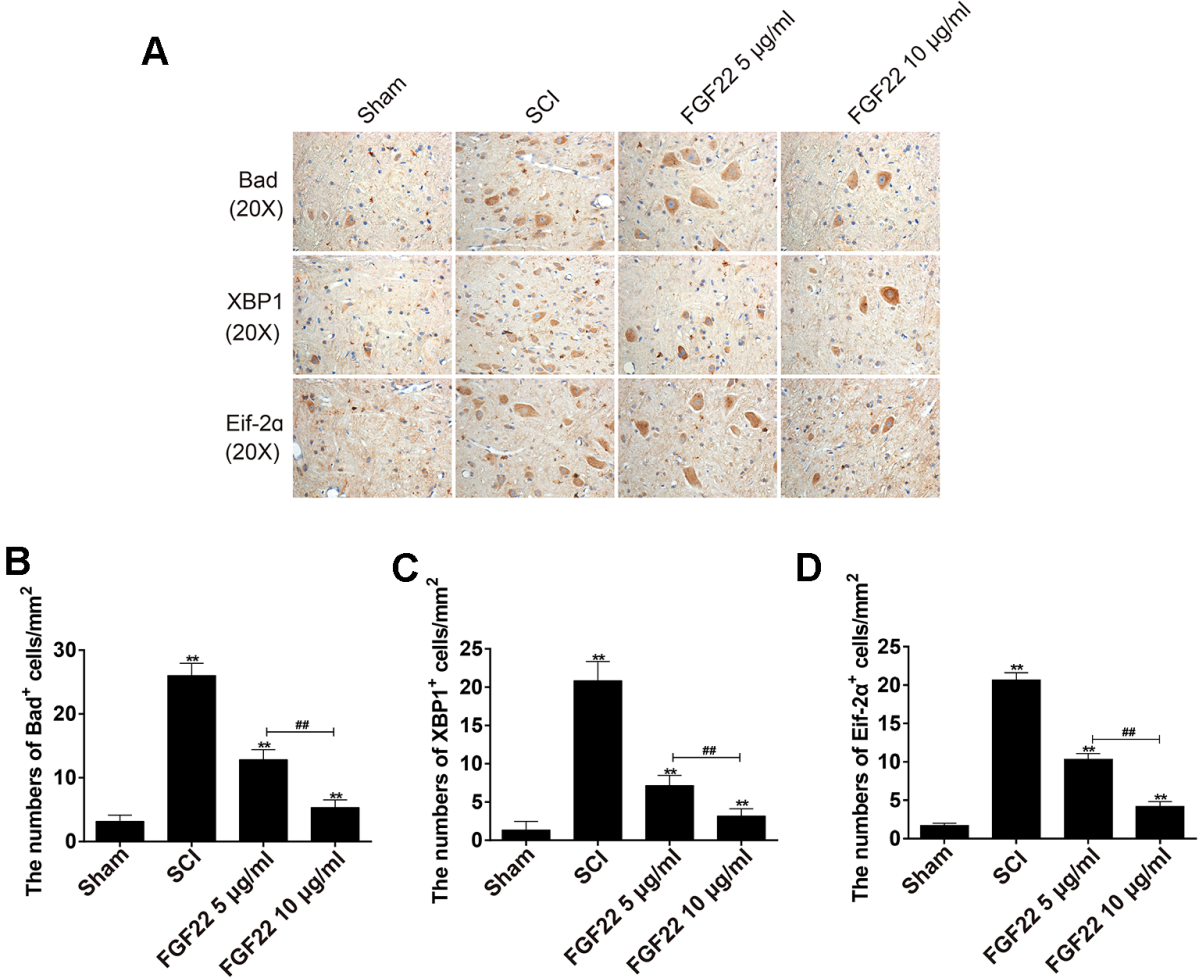
Regulation of upstream and downstream signals is important for the protection of FGF22. **(A)** Immunohistochemistry for Bad, XBP1, and Eif-2α in the sham, SCI, 5 μg/ml FGF22 and 10 μg/ml FGF22 groups. **(B**–**D)** Analysis of positive cells in immunohistochemistry of crosscutting. “**” represent *P* < 0.01 versus the sham group or SCI group, “^##^” *P* < 0.01 indicating statistically significant. Data are the mean values ± SEM, *n* = 6.

Eif-2α was an upstream factor of CHOP, which promoted JNK signaling and inhibited AKT signaling to promote apoptosis. XBP1 promoted apoptosis by activating JNK signaling (Li et al., 2019). Bad was the downstream factor of CHOP, CHOP-ERO1α-caspase-dependent apoptosis signaling pathway activates mitochondrial-mediated apoptosis signaling pathway by up-regulating BAD and caspase-3 and down-regulating BCL-2/BAX ratio (Chen et al., 2019). To determine that the regulation of upstream and downstream signals was important for protecting FGF22, we measured the expression of Bad, XBP1, and Eif-2α associated with endoplasmic reticulum stress by immunohistochemical staining. As shown in **Figure 6**, the expression of Bad, XBP1 and Eif-2α increased in SCI and were significantly suppressed by FGF22. In addition, the 10 μg/ml FGF22 group had fewer positive spots than the 5 μg/ml FGF22 group. This indicates that within a certain range, 10 μg/ml FGF22 can better inhibited apoptosis and promoted the recovery of SCI (**Figures 6A–D**).

### Recovery of Hindlimb Function in Rats Treated With FGF22 Inhibiting ER Stress-Induced Apoptosis

**Figure 7:**
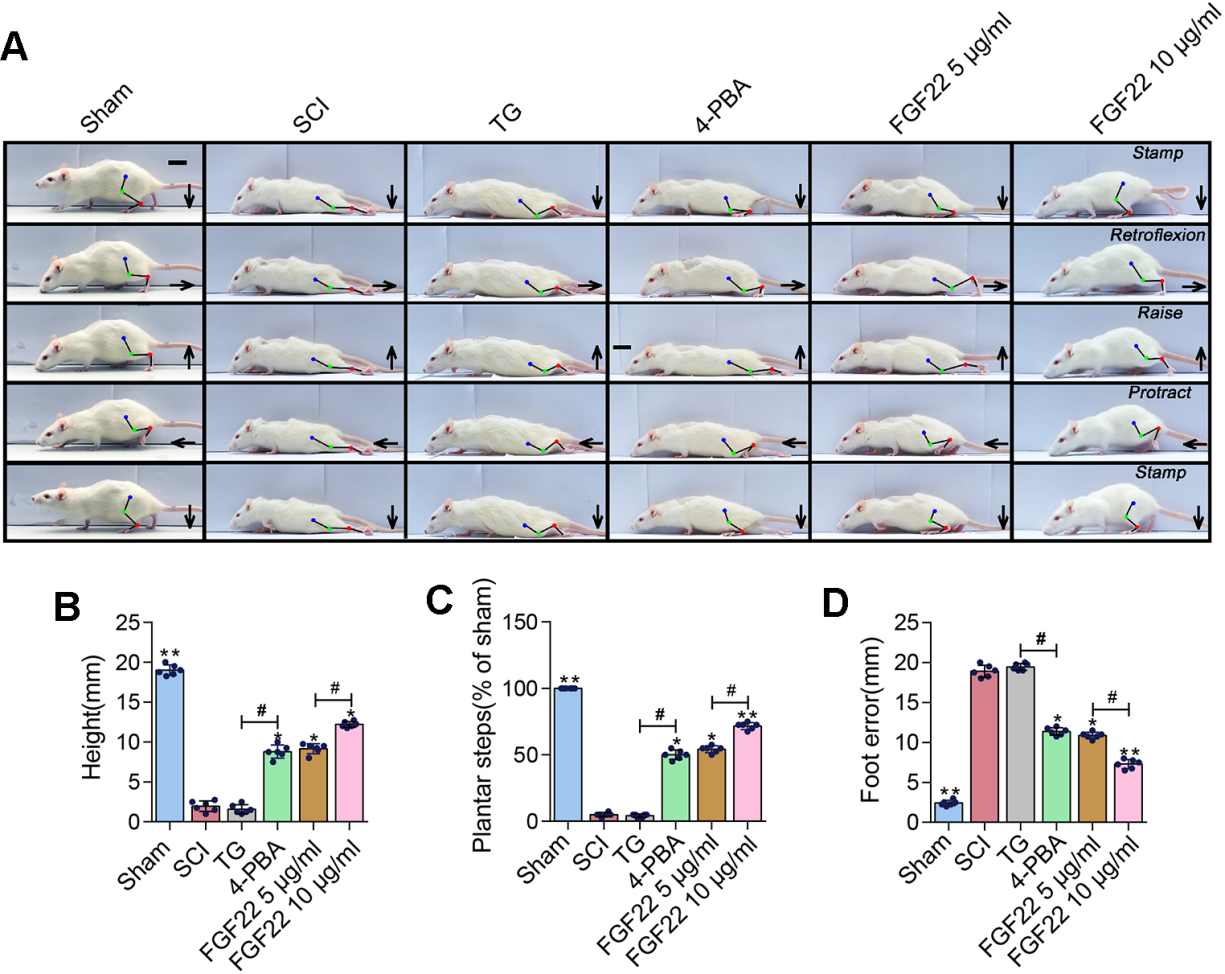
FGF22 improved hindlimb function SCI rats *via* the inhibition of ER stress-induced apoptosis. **(A)** Video image sequence of rat walking posture of the sham group, SCI group, TG group, 4-PBA group, 5 μg/ml FGF22 group and 10 μg/ml FGF22 group in spinal cord injury, at 14 days after SCI. Lines and dots were used to mark the ankle, knee and hip joint, and foot movement was shown by arrows, scale bar = 500 μm. **(B)** Body height for each rat of the six groups. “*” and “**” represent *P* < 0.05 or *P* < 0.01 versus the SCI group, “^#^” represent *P* < 0.05. Data are the mean values ± SEM, *n* = 6. **(C)** Number of successful hind-limb steps for each rat of the six groups. “*” and “**” represent *P* < 0.05 or *P* < 0.01 versus the SCI group, “^#^” represent *P* < 0.05. Data are the mean values ± SEM, *n* = 6. **(D)** Foot-placement error for each rat of the six groups. “*” and “**” represent *P* < 0.05 or *P* < 0.01 versus the SCI group, “^#^” represent *P* < 0.05. Data are the mean values ± SEM, *n* = 6.

TG was an endoplasmic reticulum stress agonist and 4-PBA was an endoplasmic reticulum stress inhibitor (Rellmann et al., 2019). Adding these two groups as the comparison can showed that inhibition of endoplasmic reticulum stress can promoted the recovery of SCI. The FGF22 groups showed lower index of foot error and higher index of height and plantar steps which indicated preferable recoveries (**Figure 7A**). The 4-PBA group also showed better functional recovery, this illustrated that inhibiting ER stress promoted the recovery after SCI. It can also be obtained from video data that 10 μg/ml FGF22 group showed signiﬁcantly recovery of hindlimb function of rats after SCI. It had least foot error, highest index of height and plantar steps than the other two groups (**Figures 7B–D**). Meanwhile, the TG treated group showed no obvious improvement. This suggested that ER stress has a negative effect on functional recovery after SCI. Inhibiting ER stress-induced apoptosis effectively promote the hindlimb function of SCI rats.

### FGF22 Treatment Promotes Neuronal Survival and Nerve Regeneration

**Figure 8:**
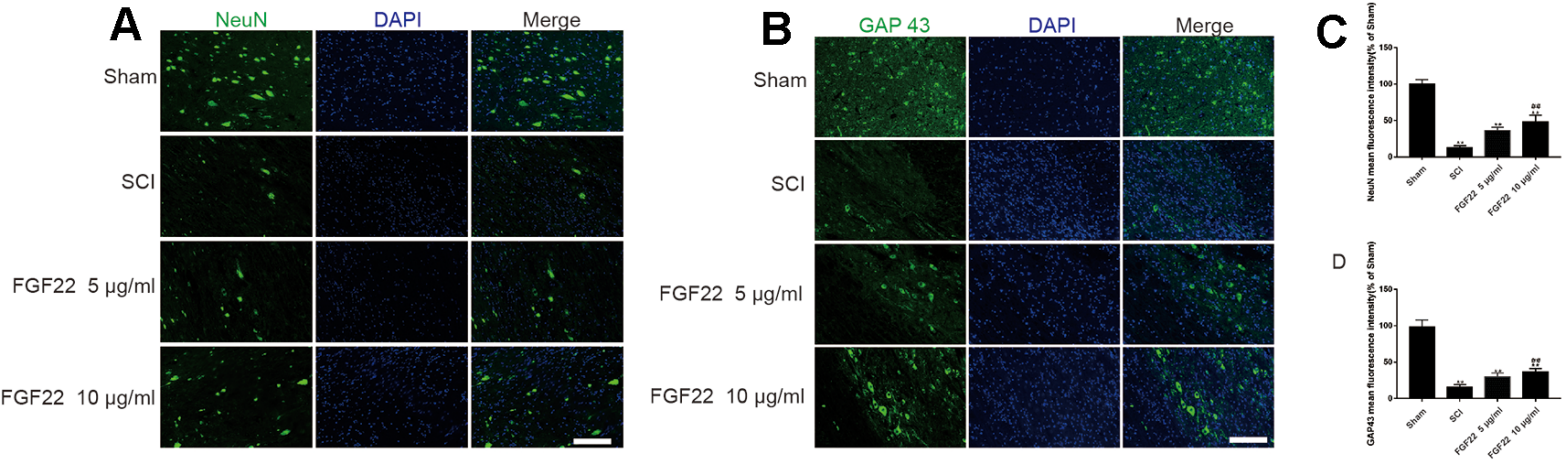
FGF22 increased the level of NeuN and GAP43 in spinal cord lesions. **(A)** NeuN staining of the sham, SCI, 5 μg/ml FGF22, and 10 μg/ml FGF22 groups. The bright green dots in the right column are considered positively stained neurons, the nuclei are labeled by DAPI (blue). Magnification was ×20. **(B)** Immunofluorescence staining of GAP43 in spinal cord lesions; the nuclei are labeled by DAPI (blue). The bright green dots in the right column are considered positive staining for GAP43, magnification was ×20. **(C**, **D)** Analysis of positive cells in immunofluorescence staining. “**” represents *P* < 0.01 versus the sham group or SCI group, “^##^”represents *P* < 0.01 10 μg/ml FGF22 than 5 μg/ml FGF22 group plays have statistical significance. Data are the mean values ± SEM, *n* = 6.

To determine the effect of FGF22 on apoptosis inhibition, NeuN was used to detect neuronal numbers. The neurons in the SCI group were largely lost, while the FGF22 treatment groups showed increases in the number of neurons, which proved that apoptosis was effectively inhibited. The higher concentration of FGF22 had more obvious protective effects on neurons. The experimental results showed an obvious enhancement of positive green fluorescence signal in the 10 μg/ml FGF22 administration group compared with the 5 μg/ml FGF22 group and SCI group (**Figures 8A, C**).

Moreover, we used GAP43 to detect the effect of FGF22 on axons. After SCI, axons break and are difficult to regenerate. The number of axons is significantly increased with the injection of FGF22. The experimental results showed an obvious enhancement of positive green fluorescence signal in the axons in the 10 μg/ml FGF22 administration group compared with the 5 μg/ml FGF22 group and SCI group (**Figures 8B, D**). This indicates that FGF22 can promote axonal regeneration after spinal cord injury, restore the nerve conduction pathway and promote functional recovery.

## Discussion

After SCI, the injury site forms a microenvironment of ischemia, hypoxia and inflammatory infiltration (Abbott et al., 2010). After the neurons are stimulated by the unbalanced microenvironment, protein folding errors, unfolded protein aggregation, and Ca^2+^ balance disorders occur in the ER (Soboloff and Berger, 2002; Montague et al., 2014). This state is called endoplasmic reticulum stress. ER stress can trigger a series of physiological changes, and the misfolded proteins accumulated in the endoplasmic reticulum are processed, which is beneficial to the recovery of cell function (Lin et al., 2005). This process is called the unfolded protein response (UPR) and is a cytoprotective mechanism that restores ER homeostasis under cell pressure (Grootjans et al., 2016). However, if the UPR is beyond the control of protein folding and uncompensated, it will activate the apoptotic signaling pathway (Ohri et al., 2011). Unfolded proteins are stored in the ER and induce activation of PERK/ATF6 signaling pathways. The activation of this pathway results in upregulated expression of chaperones including GRP78, followed by activation of caspase-12 and other apoptotic proteins, such as CHOP, to induce cell apoptosis (Nakagawa et al., 2000; Li et al., 2016).

When ER stress occurs, ER oligomerization occurs, and phosphorylation leads to phosphorylation of Eif-2α, which forms the p-eIF2α pathway, inhibits and inhibits mRNA transcription, and reduces protein accumulation (Sozbilen et al., 2018). In addition to Eif-2α, XBP1 is also activated in the transcription of the ER chaperone gene (Wang et al., 2013). The following is the binding of the general transcription factor nuclear factor Y to the CCAAT part of the ERSE, which leads to the activation of ER chaperone gene transcription (Ubeda and Habener, 2000; Shifman et al., 2008).

Previous studies have indicated that SCI causes a large number of neuronal deaths, axonal rupture, and disruption of nerve conduction pathways (Zhou et al., 2017; Anderson et al., 2018). Among them, the loss of neurons has the most direct relationship with motor dysfunction (Liu et al., 2015). There is increasing evidence indicating that ER-stress-activated pathways may play a significant role in promoting cell death caused by apoptosis in CNS injury diseases (Byrnes et al., 2007; Ogawa et al., 2007). Therefore, a variety of mechanisms, such as disturbed ionic oxidative stress, homeostasis, ER stress, and inflammatory response, participate in the progression of the second pathological process of SCI (Rami and Kogel, 2008). Since neurons are difficult to regenerate, the best solution for neuronal loss is to protect neurons and reduce their apoptosis. Therefore, inhibiting this process to protect neurons from apoptosis might be an ideal therapeutic strategy for SCI.

Many therapeutic interventions utilizing growth factors, such as basic fibroblast growth factor (bFGF), nerve growth factor (NGF), and vascular endothelial growth factor (VEGF), have been confirmed to promote functional recovery after SCI (Rathbone et al., 1999; Zhu et al., 2016; Anderson et al., 2018). However, these factors are usually applied to promote angiogenesis, neuronal proliferation and differentiation but are rarely used to inhibit neuronal apoptosis.

In this research, we demonstrated that ER stress-induced apoptosis participates in the response to SCI and the levels of proteins activated by apoptosis, including CHOP, GRP78, and caspase-12, were significantly increased by SCI. As intermediate signaling molecules of the ER stress pathway, these proteins mediate apoptosis through various pathways such as the IRE-1-CHOP pathway, ATF6-CHOP pathway, and PERK-elF2α-ATF4-CHOP pathway, which affect the expression and function of downstream proteins.

As an important promoter of circuit remodeling in the injured spinal cord, FGF22 treatment inhibited the expression of protein activated by ER stress and improved motor function and SCI recovery (Singh et al., 2012). Previous studies also showed that genetic destruction of the FGF22 signal not only reduces the number of new axons formed in the injured loci but also changes the molecular organization of the forming axons (Terauchi et al., 2010). Therefore, treatment of injured spinal cord with FGF22 can effectively promote axonal regeneration and functional recovery (Huang et al., 2017). The experimental results showed that apoptosis signaling molecules in the FGF22 treatment group were inhibited, which indicated that FGF22 can effectively inhibit endoplasmic reticulum stress, reduce neuronal apoptosis and play a crucial role in neuronal protection.

In conclusion, our research demonstrated that FGF22 plays a role in protecting neurons of SCI rats, promoting functional recovery after SCI by reducing apoptosis induced by ER stress and promoting axon regeneration [46]. To further prove that FGF22 indeed functions through the ER stress pathways to promote SCI recovery, we will use Chop knockout SD rats and further verify our experimental results.

## Data Availability Statement

All datasets generated for this study are included in the article/supplementary material.

## Ethics Statement

The animal study was reviewed and approved by the Animal Care and Use Committee of Wenzhou Medical College.

## Author Contributions

SZ, MiC, MeC, and JY coordinated and carried out most of the experiments and data analysis and participated in drafting the manuscript. MeC to participate in the data. YY, QW, HD, and LB provided technical assistance, including drawing and tabulating. SZ, MiC, and JY carried out data analysis and revised the manuscript. FM, WN, and KY supervised the project and experimental design and provided financial support. SZ and MiC wrote the main part of the paper. All authors read and approved the final manuscript.

## Funding 

This work was partly supported by a research grant from the National Natural Science Foundation of China (81802235), Zhejiang Experimental Animal Science and Technology Project of China (2018C37112), Zhejiang Provincial Natural Science Foundation of China (LY17H060009, Y17H060051) and Wenzhou Basic Science Research Plan Project (Y20180033), which in part supported SZ as a visiting scholar at UWA. SZ made mutual collaborative visits for the purpose of this study.

## Conflict of Interest

The authors declare that the research was conducted in the absence of any commercial or financial relationships that could be construed as a potential conflict of interest.
